# Five-Year Follow-Up of Repeated Intravitreal Bevacizumab for Macular Edema in a Pediatric Patient with Retinal Arteriovenous Malformation and Excellent Vision

**DOI:** 10.1155/2023/5693657

**Published:** 2023-10-25

**Authors:** Andrew R. Miller, Ivan J. Lee, Zachary C. Wright, Monique Leys

**Affiliations:** West Virginia University Eye Institute, 1 Medical Center Drive, Morgantown, WV 26506, USA

## Abstract

We report a case of a 9-year-old girl presenting with unilateral retinal arteriovenous malformation (AVM) with symptomatic macular edema. Over 5 years of follow-up includes optical coherence tomography (OCT), fundus photographs, and fluorescein angiography at baseline and at follow-up. Systemic and neurologic workup was completed and negative for intracranial AVM. Vision has correlated with macular edema, ranging from 20/20 to 20/80. The patient has received nine injections of intravitreal bevacizumab and has not required an injection for the last couple of years. Follow-up is ongoing.

## 1. Introduction

Retinal arteriovenous malformations are a rare condition with a wide variety of presentations depending on location and severity [1–3]. In 1973, Archer et al. presented six cases of retinal arteriovenous communications and separated them into three groups [1]. Our patient would likely fit the original group 2, characterized by “direct arteriovenous communication without the interposition of capillary or arteriolar elements” and with a hyperdynamic flow pattern in the involved vessels and possibility of decompensation and possible sequelae such as retinal edema, exudates, and hemorrhage. Group 1 was characterized by an arteriolar or abnormal capillary plexus, generally localized to one quadrant or sector of the retina, and generally “protected” against decompensation. Group 3 was characterized by more severe vascular changes leading to severe vision impairment and was more consistent with Wyburn-Mason's syndrome. In 1989, Mansour et al. described complications related to retinal arteriovenous communications, including intraretinal macular hemorrhage, central and peripheral retinal vein occlusions, neovascular glaucoma, and vitreous hemorrhage [3].

## 2. Case Presentation

**Figure 1:**
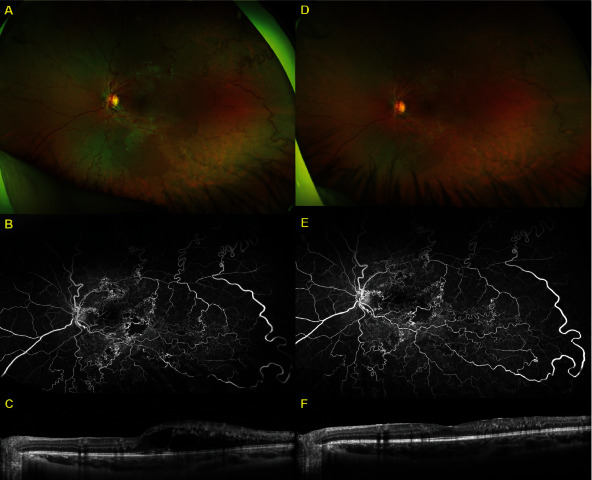
Widefield fundus photos (A, D), fluorescein angiograms (B, E), and optical coherence tomography B-scans through fovea (C, F) at initial presentation (A–C) and at >3-year follow-up (38 months), also 13 weeks after the seventh intravitreal injection of bevacizumab (D–F). Note retinal arteriovenous malformation affecting all four quadrants, with intraretinal hemorrhage on presentation and areas of macular ischemia. Retinal vasculature and ischemia appear largely unchanged through this follow-up interval.

**Figure 2:**
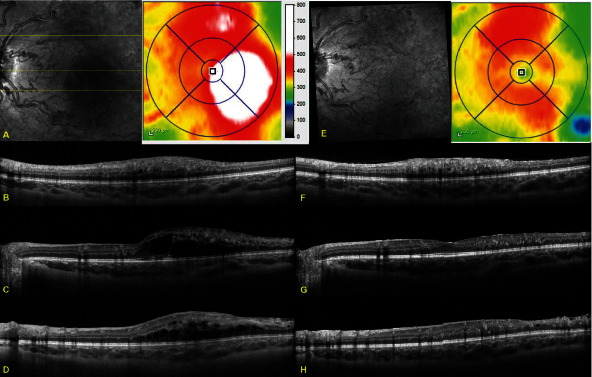
Orientation infrared images and retinal thickness maps (A, E) and optical coherence tomography B-scans through superior macula (B, F), fovea (C, G), and inferior macula (D, H) at initial presentation (A–D) and at >3-year (38 months) follow-up, also 13 weeks after seventh intravitreal injection of bevacizumab (E–H). Attention is drawn to foci of perifoveal macular edema temporally and superiorly, disorganization of retinal inner layers, engorged and tortuous retinal vessels, peripheral retinal thinning, and good response of macular edema to intravitreal bevacizumab therapy.

A healthy 9-year-old girl was referred to a retina specialist for vision changes in the left eye and headache. Best corrected Snellen visual acuity was 20/20 in the right eye and 20/60-2 in the left eye. No relative afferent pupillary defect was present, and anterior segment examination and intraocular pressure were unremarkable in both eyes. Dilated exam of the retina was normal in the right eye, but diffuse arteriovenous malformation in macula and all four quadrants was noted in the left retina (Figure 1). The optic nerve appearance was normal. No soft or hard exudates were observed, though one intraretinal hemorrhage was noted inferiorly to the inferior arcade. Optical coherence tomography (OCT) revealed normal retinal layers and contour in the right eye, but the left eye had significant cystoid edema temporal to the fovea with disorganization of retinal inner layers associated with dilated and abnormal vessels superiorly, temporally, and inferiorly with relative sparing of the nasal macula (Figure 2). Widefield fluorescein angiography revealed areas of arteriovenous malformation with multiple areas of macular and peripheral capillary dropout but no neovascularization (Figure 1). Initial treatment was attempted with topical dorzolamide, but no improvement was noted after 4 weeks of use. After insurance approval and discussion with the patient and her parents, intravitreal bevacizumab (1.25 mg/0.05 mL) was utilized two weeks after presentation and administered in the clinic. Vision improved from 20/70 to 20/40 at two weeks later and 20/30 at two months after treatment. The improved visual acuity correlated with improvement in macular edema on OCT.

Edema began to recur at four months, and she received a second injection. After this, she was noted to have a cotton wool spot in the superior macula at a 2-month follow-up. She continued to have PRN bevacizumab injections. She was noted to have a macular dot-blot hemorrhage between five and six months after her fifth injection, along with a decrease in vision to 20/80+1 and new subretinal fluid at that visit. This subretinal fluid resolved with her sixth injection. Repeat widefield fluorescein angiography was performed over three years after her initial angiography and three months after her seventh injection. No significant changes were appreciated in retinal vasculature or capillary dropout, and no retinal neovascularization was present. Thus far, macular edema and visual acuity have continued to improve with injections, and she has received nine intravitreal injections of bevacizumab at approximately four- to seven-month intervals (Table 1). The patient has not required an injection in the last couple of years. No laser therapy has been administered. Visual acuity during follow-up has ranged from 20/20 to 20/80 and correlated with the amount of macular edema.

Our patient was noted to have a history of 6-week prematurity with brief NICU stay and no oxygen. After diagnosis of her retinal vascular malformation, neurology consultation was obtained due to concern for association with intracranial vascular malformations, and MRI and MRA were performed and normal. She had no facial or cutaneous symptoms indicative of the Wyburn-Mason or Bonnet-Dechaume-Blanc syndrome. Family history was positive for epilepsy but negative for known ophthalmic conditions other than refractive error. Laboratory evaluation was also performed. Basic plasma chemistries and blood counts were unremarkable. Full gene analysis was negative for the Von Hippel-Lindau disease. An inherited retinal disorder panel of 293 genes (Invitae Corporation, San Francisco, CA) was performed on a saliva sample and was positive for heterozygous mutations in two genes of uncertain significance, *PITPNM3* and *VCAN*, but negative for all other tested markers. She remains a healthy young woman and is currently in middle school, particularly enjoying sports such as basketball.

## 3. Discussion

Archer reported 80 published cases of retinal AVMs in 1973, and many more have been published since [1]. As with many retinal pathologies, the advent of antivascular endothelial growth factor (VEGF) agents has revolutionized management. Review of the literature reveals at least ten cases of intravitreal anti-VEGF agents for complications [4] or macular edema [5] from retinal arteriovenous malformations dating back to 2010 and at least one case of posterior subtenon triamcinolone [6]. Focal laser [7] has also been utilized for macular edema. Many of the reported cases presented well into adulthood, and reports of treatments tend to report stabilization after one and no more than three anti-VEGF injections, although few have long-term follow-up. Other patients have very poor vision on presentation due to macular or optic nerve involvement.

In this case, a close five-year follow-up of an isolated retinal arteriovenous malformation is presented. This patient has received nine injections of intravitreal bevacizumab for symptomatic macular edema, and four to seven months between injections (Table 1) seems a good interval from our experience with this patient. In the last couple of years, the patient has not developed macula edema, suggesting possible hormonal influence on vascular integrity in adolescent patients with AVM. In this case, hemorrhages and cotton wool spots have been noted throughout treatment. Despite keeping macular edema at bay, bevacizumab has not improved nonperfusion. Nevertheless, no significant worsening of ischemia or vessel sclerosis was caused by treatment during follow-up (Figure 1). Vision in the affected eye remains excellent (20/20), and the main concern going forward is preservation of good visual function in this young patient. While monitoring for the aforementioned vascular complications, we are considering therapies such as focal laser or peripheral photocoagulation. Despite good therapeutic response to bevacizumab and experience with bevacizumab in pediatric patients, VEGF-trap could also be considered.

As ophthalmologists with experience treat VEGF-driven retinal vascular diseases, the use of anti-VEGF agents to treat macular edema has become almost reflexive. In arteriovenous malformations, aberrant vascular development leads to altered intravascular dynamics in abnormal vessels. As Archer et al. reported, some of these malformations can be relatively protected against decompensation [1]. Others are prone to decompensation and a variety of other complications [3]. The precise cause of macular edema in AVMs has not been established; however, there appears to be some threshold beyond which retinal vessels begin to leak, perhaps through tight junctions in the endothelium of already faulty anastomosing vessels. The contribution of VEGF to edema in retinal AVMs is unclear, but bevacizumab's VEGF inhibition presumably leads to decreased vascular permeability. For the continued visual function and overall health of this young patient, we expect that anti-VEGF therapy will remain the mainstay of her treatment plan even if other therapies (e.g., laser) are utilized.

## 4. Conclusions

Given the variety of presentations and complications associated with retinal arteriovenous malformations, astute therapeutic decisions with appropriate systemic evaluation will continue to be paramount. In this case, we demonstrate a consistent response to bevacizumab and intermittent resolution of macular edema where observation has always resulted in worsening macular edema. Bevacizumab is effective in a PRN treatment approach for macular edema due to retinal AVM.

## Figures and Tables

**Table 1:** Dates of intravitreal bevacizumab injection with intervals in months. Last injection was 5/13/21, and the patient has not required injection for the last 2 years.

Number	Date	Interval (months)
1	6/6/17	
2	10/19/17	4.5
3	4/4/18	5.5
4	10/31/18	7
5	3/26/19	4.9
6	9/12/19	5.6
7	4/22/20	7.4
8	10/29/20	6.3
9	5/13/21	6.5

## Data Availability

All case-related data are encrypted and stored at the institution.
